# Promoting Flourishing at Work: Innovations in Positive Work and Organisational Psychology

**DOI:** 10.3390/bs16040555

**Published:** 2026-04-08

**Authors:** Rona Hart, Dan Hart

**Affiliations:** 1 School of Psychology, University of Sussex, Falmer, Brighton BN1 9RH, UK; 2 Birmingham Business School, University of Birmingham, 116 Edgbaston Park Road, Birmingham B15 2TY, UK; d.hart@bham.ac.uk

## 1. Introduction

Work occupies a central place in contemporary life. For many individuals, it is not merely a means of securing material resources but also a source of identity, purpose, personal development, and social connection. Work structures daily routines, shapes self-understanding, and contributes substantially to happiness and fulfilment. At the same time, the workplace can also be a source of tension, conflict, and distress. In the 21st century, attending to occupational wellbeing is therefore no longer a peripheral concern but a strategic imperative for sustainable organisations.

Positive Work and Organisational Psychology (or Positive Work and Organisations) has emerged as a dynamic and increasingly influential field of scholarship and practice concerned with how work can be designed, led, and experienced in ways that promote human flourishing while also supporting sustainable organisational performance and productivity. The growth of the field reflects a recognition that work often falls short of being meaningful, engaging, healthy, or even tolerable. Gallup’s (2022) global report on the state of work captures this bluntly, concluding that for most people “work sucks” (p. 2). Gallup estimated that only 23% of employees are engaged, whereas 59% are not engaged (quiet quitting) and 18% are actively disengaged. Wellbeing indicators are similarly concerning: across 160 countries, only 33% of employees are classified as “thriving”, with the remainder described as “struggling” or “suffering” (Gallup, 2022). Occupational stress also remains pervasive, with 44% reporting persistent stress and 51% demonstrating intentions to leave their jobs (Gallup, 2023). Although these figures have shown modest improvement over earlier decades, they reveal a persistent gap between what work currently offers and what it could enable under more supportive organisational conditions.

Set against this bleak backdrop, Positive Work and Organisations offers a timely corrective by examining the conditions under which employees and organisations can function well and flourish. It conceptualises workplaces as more than sites of economic production; they are social and developmental contexts that shape identities, cultivate capabilities, influence life trajectories, and inform beliefs about the self and society. At its best, work can provide structure, opportunities for learning and achievement, autonomy, development, social connection, recognition, and respect. For many people, it is also a key source of meaning, purpose, and contribution beyond the self. Because work is so closely tied to fundamental human needs and values, its quality has profound implications for wellbeing.

This Special Issue positions Positive Work and Organisations as both a conceptual reorientation and an applied agenda aimed at bringing organisational practices and outcomes into closer alignment with employee wellbeing. At the same time, as demonstrated below, the field is facing a critical junction in its trajectory, one that can determine its fate.

## 2. Conceptualising Positive Work and Organisations

Positive Work and Organisations integrates insights from Positive Psychology and Management scholarship and is defined as the study and application of psychological mechanisms that promote employee and organisational flourishing (Warren et al., 2017). As an applied science, it is oriented towards “developing strategies to enhance positive functioning and creating evidence-based intervention strategies to help facilitate individual wellbeing and organisational flourishing” (van Zyl et al., 2024, p. 700).

The term Positive Work and Organisations functions as an umbrella concept encompassing three overlapping sub-domains:

- **Positive Organisational Psychology (POP):** This sub-domain refers to the scientific study of positive subjective experiences in the workplace. It centres on employees and the interface between individuals and their organisations, with a strong applied emphasis on developing interventions that enhance quality of working life (Donaldson & Ko, 2010). It therefore addresses topics such as occupational wellbeing, positive leadership, job satisfaction, engagement, motivation, positive relationships, and meaningful work.
- **Positive Organisational Behaviour (POB):** This strand is defined as “the study and application of positively oriented human resource strengths and psychological capacities that can be measured, developed, and effectively managed for performance improvement in today’s workplace” (F. Luthans, 2002, p. 59). It focuses on individual psychological resources associated with wellbeing and performance, including strengths, hope, optimism, resilience, and prosocial behaviour (F. Luthans, 2002).
- **Positive Organisational Scholarship (POS):** This sub-field is described as “the study of that which is positive, flourishing, and life-giving in organisations” (K. S. Cameron & Caza, 2004, p. 731). It focuses on organisational characteristics, processes, and outcomes and on the interpersonal and structural dynamics that emerge within organisational life (K. S. Cameron & Caza, 2004). Typical topics include organisational virtuousness, positive deviance, and appreciative cultures (K. S. Cameron et al., 2003). Its primary emphasis is on achieving work-related outcomes through positive means.

As these descriptions indicate, the three sub-domains overlap considerably, which has contributed to conceptual ambiguity. To address this, Warren et al. (2017) proposed the umbrella term Positive Work and Organisations (PWO) as a more coherent integrative framework. The purpose of PWO is “to encourage dialogue across subfields and serve as a clearinghouse for best practices in Positive Psychology theory building and practice” (Donaldson et al., 2019, p. 114).

Two concepts are particularly central to the field and warrant closer clarification:

- **Flourishing employees:** Within the occupational domain, flourishing refers to a state of optimal occupational wellbeing in which individuals are satisfied with their work, engaged in what is morally worthwhile, and functioning optimally both in their work roles and across the interface between work and other life domains. Flourishing employees possess a positive mental state and engage in behaviours that contribute to their organisations (Seligman, 2012).
- **Flourishing organisation:** A flourishing organisation is one that embeds positive values and practices into its day-to-day functioning. Such organisations achieve high performance and profitability by bringing out the best in employees, enabling them to engage, grow, and flourish. They also leverage employees’ strengths and talents by designing work that is meaningful and empowering (K. Cameron, 2012).

## 3. The Remit of Positive Work and Organisations

A broad range of topics falls within the remit of PWO (Martin-Del-Rio et al., 2021; Warren et al., 2017; Donaldson & Ko, 2010; F. Luthans & Youssef-Morgan, 2020). These may be grouped into the following themes:

- **Employee wellbeing:** This area examines wellbeing across multiple interconnected domains, including physical, mental, social, occupational, and financial wellbeing. Within occupational wellbeing specifically, it considers how employment conditions, career trajectories, and everyday work experiences shape quality of life and functioning. Applied work in this area focuses on identifying risks and protective factors, strengthening resources, and developing interventions such as stress management, recovery practices, burnout prevention, and evidence-informed workplace wellness initiatives.
- **Happiness at work:** This theme examines the conditions shaping satisfaction and positive affect at work and how these influence outcomes such as performance and organisational commitment. It is often linked to the happy–productive employee model (Cropanzano & Wright, 2001), which proposes that employees who are happier at work tend to perform better because positive affect supports motivation, persistence, and constructive work behaviour.
- **Positive organisational behaviours:** This theme encompasses research on individual and collective behaviours that foster constructive and supportive work environments, including optimism, resilience, self-efficacy, and self-regulation. It also includes prosocial and relational behaviours that strengthen teams and organisations, such as cooperation, organisational citizenship behaviours, kindness, and peer support.
- **Engagement and performance:** This topic examines what energises and sustains employee engagement and effectiveness, and how these translate into performance. It includes individual factors such as motivation, strengths, and work-related meaning, alongside organisational factors such as job design, the availability of resources, autonomy, supportive leadership, learning opportunities, and recognition. It also considers how these influences interact.
- **Strengths-based approaches:** These approaches emphasise the identification, development, and application of individual and team strengths. In practice, this may include strengths assessment and feedback, role crafting to align tasks with capabilities, building complementary strengths within teams, and shaping learning, coaching, and performance discussions around capability development. Closely related is **Appreciative Inquiry**, an organisational development approach that builds on existing strengths by systematically exploring successes, resources, and possibilities to envision and co-create desired futures. The underlying premise is that these practices can enhance energy, confidence, engagement, collaboration, and effectiveness while still addressing weaknesses where they constrain wellbeing or performance.
- **Meaning and purpose at work:** This area examines how people experience work as significant and worthwhile and how this sense of meaning shapes motivation, engagement, and performance. It explores where meaning comes from and how organisations can foster it through job design, leadership, and organisational narratives. Common topics include work orientations, such as understanding work as a job, a career, or a calling, and how these orientations help employees connect everyday tasks to a broader purpose.
- **Psychological Capital (PsyCap):** PsyCap refers to a set of psychological resources that support effective functioning at work. Research in this area examines how these resources shape employees’ interpretations of challenges, goal-setting, persistence through setbacks, and recovery from adversity, and how they relate to outcomes such as engagement, performance, adaptability, and wellbeing.
- **High-quality work relationships:** This domain examines how supportive, trusting, and growth-oriented workplace relationships are formed, maintained, and linked to positive outcomes. It focuses on the quality of everyday interactions among colleagues, leaders, and followers. Key topics include high-quality connections, relational energy, leader–member exchange, psychological safety, trust and fairness, inclusion and belonging, compassion and civility, social support, and constructive conflict repair. Research also considers how high-quality relationships influence wellbeing, engagement, performance, creativity, adaptability, and retention, while recognising that poor relationships can undermine health and organisational effectiveness.
- **Positive change management:** This theme focuses on how organisational change can be designed and led in ways that strengthen rather than deplete employee and organisational functioning. It examines approaches that foster psychological safety, participation, and trust; communicate a compelling purpose and clear rationale for change; and support adaptation through learning, capability-building, and adequate resources. It also considers how leaders can mobilise positive emotions, strengths, and collective efficacy during transitions; reduce uncertainty and change fatigue; and create change processes that leave organisations healthier and more resilient than before.
- **Work–life balance:** This topic examines how work intersects with other life domains and how people manage the boundaries, demands, and spillover between them. It considers individual strategies such as boundary-setting, time and energy management, and role negotiation, alongside organisational conditions such as workload, scheduling, flexibility, remote work arrangements, supportive leadership, leave policies, and cultures that respect non-work time. It also addresses outcomes such as stress, wellbeing, engagement, retention, and the extent to which work may either enrich life beyond work or generate conflict and depletion.
- **Positive leadership:** This area examines leadership approaches that cultivate healthy, high-functioning teams and constructive workplace climates, including transformational, authentic, and servant leadership. It focuses on how leaders inspire and motivate through articulating a compelling vision, modelling integrity and optimism, and fostering trust, psychological safety, and inclusion. It also addresses everyday leadership practices that bring out the best in others, such as recognition, appreciation, developmental support, and the cultivation of high-quality relationships.
- **Organisational culture and climate:** This area examines the shared values, norms, and assumptions that shape how organisations function, alongside employees’ perceptions of what is expected, supported, and rewarded in everyday working life. It also includes the **psychological contract**, understood as the implicit and subjective beliefs employees and employers hold regarding mutual obligations. Research in this domain explores how culture, climate, and psychological contracts interact to influence morale, wellbeing, trust, collaboration, learning, productivity, ethical behaviour, and retention, and how perceived contract fulfilment or breach may strengthen commitment or trigger disengagement and turnover intentions.

Taken together, these themes demonstrate PWO’s dual concern with understanding how people thrive at work and how organisations can cultivate the conditions that enable such thriving. The remit of the field spans individual experience, psychological resource development, and the relational and structural characteristics of work. This breadth reflects the field’s integrative ambition: to connect rigorous psychological theory with organisational practice and to translate that knowledge into strategies that enhance both employee functioning and organisational effectiveness.

## 4. The Development of Positive Work and Organisations

In a bibliometric review and mapping analysis, Martin-Del-Rio et al. (2021) identified PWO as one of the fastest-growing interdisciplinary subdomains within Positive Psychology. Their analysis highlighted its multidisciplinary reach and the extent to which its theories and methods have travelled beyond traditional disciplinary boundaries. This diffusion has facilitated collaboration and knowledge exchange, thereby enhancing the field’s visibility, credibility, and impact.

Despite this growth, however, there are signs that the field’s influence may be weakening. Martin-Del-Rio et al. (2021) found that the publications attracting the greatest scientific attention were concentrated between 2001 and 2011. Critics have suggested that PWO has produced relatively few genuinely transformative ideas since then and that some more recent work has generated mainly “ephemeral frontier knowledge” that does not accumulate in ways that advance theory or sustain knowledge-building (van Zyl et al., 2024, p. 701).

van Zyl et al. (2024) attributed the field’s rapid rise to a combination of strategic visibility and contextual fit. They emphasised the role of influential scholars in promoting PWO, early funding that enabled high-profile programmes and international collaboration, and wider social and political shifts that foregrounded wellbeing and agency, thereby making organisations more receptive to positive approaches to work. They also highlighted the appeal of PWO’s applied, results-oriented promise: developable strengths, positive psychological states, and healthier work environments are often framed as pathways to improved performance. The field’s traction was further amplified by the mainstreaming of constructs such as work engagement and strengths use, by endorsement and implementation through large consultancy and analytics firms, and by institutional recognition that enhanced its credibility and reach.

At the same time, several factors appear to have constrained the field’s further progression and contributed to its apparent stagnation (van Zyl et al., 2024). These include weak meta-theoretical grounding, substantial borrowing across traditions, methodological limitations, the instrumentalisation of wellbeing and meaning in service of productivity, the tendency to individualise responsibility while deflecting attention from harmful working conditions, insufficient responsiveness to changes in work structures and technology, ethical concerns, a declining pipeline of future professionals, and uneven practitioner competence. Collectively, these issues limit its theoretical and empirical development, reduce confidence in its research claims, damage the field’s reputation, and slow the uptake of ideas and interventions by practitioners and stakeholders.

In response to these concerns, van Zyl et al. (2024) argued that PWO is entering a second wave shaped by both scholarly stagnation and the rapidly changing nature of work. This second wave explicitly embraces technological transformation. It is defined as an evidence-based, data-driven approach that uses technological advances and human-centred design to enhance positive characteristics of individuals and organisations in the service of functioning, wellbeing, and performance. PWO 2.0 expands the field’s remit beyond conventional organisational settings to include digital and decentralised workforces, including contexts involving artificial intelligence companions. It also calls for stronger research designs and more purposeful use of machine learning, big data analytics, natural language processing, and passive assessment to capture work experiences in real time.

This second wave also implies a shift in professional practice and identity. Practitioners are positioned less as facilitators alone and more as developers, analysts, and data-driven technologists who help design and govern environments that support wellbeing. It also encourages multilevel thinking, expanded interdisciplinarity, culturally sensitive and inclusive perspectives, and deeper stakeholder collaboration to ensure that the science remains relevant and usable in real organisational contexts.

Overall, these developments suggest that the first wave of PWO achieved rapid expansion through strategic promotion, strong socio-economic resonance, and an attractive applied promise. However, the past decade has exposed vulnerabilities in theoretical novelty and cumulative progress. The second-wave agenda may therefore be understood as both a corrective and an opportunity: an attempt to renew the field’s intellectual momentum by aligning its concepts, methods, and professional capabilities with digitally mediated work realities.

## 5. Applied Interventions and Their Impact

In their systematic review of Positive Psychology interventions in organisational contexts, Donaldson et al. (2019) identified five broad categories of interventions commonly implemented in workplaces:

- **Wellbeing interventions:** These range from environmental and ergonomic provisions to physical activity programmes, mindfulness sessions, health and safety training, resilience-building, stress management, and self-care practices. Most are delivered in group formats and are intended to encourage employees to adopt and sustain behaviours that support wellbeing. Such interventions typically target individuals’ attitudes and behaviours rather than work conditions directly. As a result, broader environmental change is more likely to occur indirectly and gradually through the cultivation of a workplace culture that normalises wellbeing concerns. Research indicates that wellbeing interventions can reduce absenteeism and turnover intentions while improving job satisfaction (Boehm & Lyubomirsky, 2008; Layous et al., 2013). This aligns with the happy–productive employee thesis, which proposes that happier employees tend to perform better than those who are less happy (Cropanzano & Wright, 2001);
- **Psychological Capital (PsyCap) interventions:** These interventions focus on developing four psychological resources (F. Luthans & Youssef-Morgan, 2017):
  **Self-efficacy:** Confidence in one’s ability to succeed in challenging work tasks;
  **Optimism:** Positive expectations regarding one’s work and the organisation’s future;
  **Hope:** The capacity to generate goals and pathways and to reroute pathways when obstacles arise;
  **Resilience:** The ability to recover, adapt, and continue functioning effectively following adversity. Avey et al. (2010) reported that PsyCap interventions are associated with a range ofdesirable outcomes, including improved job performance, higher engagement, and more organisational citizenship behaviours. However, consistent with earlier critiques, PsyCap interventions typically focus on developing individuals rather than altering organisational systems or working conditions. That said, such interventions have also been implemented with leaders (B. C. Luthans et al., 2014; Pitichat et al., 2018), and findings suggest that strengthening leaders’ PsyCap may generate wider ripple effects by shaping culture and everyday organisational practices.
- **Strengths interventions:** Peterson and Seligman’s (2004) Values in Action (VIA) framework and its inventory of Character Strengths and Virtues, discussed earlier in this paper, has become one of the most widely used strengths-based tools in workplace contexts. In organisational applications, strengths interventions are intended to help employees identify their signature strengths, develop them further, and use them more intentionally in their work. Evidence suggests that such interventions can enhance employee wellbeing, performance, engagement, and experiences of meaningful work. They are also commonly integrated into leadership development programmes and coaching practice (MacKie, 2014).
- **Gratitude interventions:** Wood et al. (2010) conceptualised workplace gratitude as “noticing and appreciating the positive in one’s work life” (p. 891). Gratitude interventions involve structured and intentional activities designed to cultivate gratitude as a habitual orientation in the workplace. Research suggests that gratitude-based interventions can enhance employee wellbeing, happiness, and health while also strengthening workplace relationships and promoting prosocial behaviours (Wood et al., 2010; Boggiss et al., 2020).
- **Job crafting interventions:** Job crafting refers to a self-directed process through which employees modify aspects of their work in order to improve the fit between job demands and their own needs, abilities, interests, and strengths (Wrzesniewski & Dutton, 2001). Evidence suggests that when employees are able to exercise greater control over important features of their roles, they can better align work demands with personal strengths, often with benefits for performance, wellbeing, and work engagement (Tims et al., 2022). Unlike many interventions that primarily target internal psychological states, job crafting alters what employees do at work, thereby creating the possibility of incremental, bottom-up organisational change.

Donaldson et al. (2019) identified several broader patterns in the effects of these interventions. Although effect sizes varied substantially across studies, most fell within the small-to-moderate range. Overall, workplace interventions were associated with improvements in outcomes such as wellbeing, engagement, leader–member exchange, self-esteem, workplace trust, forgiveness, prosocial behaviour, leadership, and calling. Notably, however, they did not produce significant improvements in performance.

Taken together, this body of evidence suggests that PWO interventions are particularly effective in strengthening psychological and relational outcomes rather than generating direct gains in performance. One implication is that many such interventions operate through gradual and indirect pathways that may take time to translate into measurable productivity outcomes or may be constrained by job design, workload, and broader organisational systems. This points to a key conclusion: resource-building interventions are valuable, but their effects are likely to be maximised when paired with changes to the work environment so that individual gains are not undermined by structural barriers and can accumulate into sustained organisational benefit.

## 6. An Overview of Published Articles

The papers in this Special Issue collectively offer a compelling picture of the current state of scholarship on Positive Work and Organisations. As a body of work, they show that the field continues to develop in both conceptual breadth and practical relevance. Across diverse settings, occupations, and national contexts, the contributions reinforce a central proposition: positive organisational life is not produced by isolated interventions or individual dispositions alone, but through the ongoing interaction of organisational structures, leadership practices, personal resources, and meaningful work experiences.

One of the clearest themes to emerge across the issue is the importance of relational and organisational conditions in enabling positive forms of employee behaviour. Several papers show that employees are more likely to engage in constructive, discretionary, and proactive behaviours when they work in environments characterised by ethical guidance, developmental support, and psychological enablement. Su and Hahn’s (contribution 1) findings on the ways in which ethical leadership promotes organisational citizenship behaviours, Katsaros’s (contribution 2) analysis of leaders’ transformational, inclusive, and adaptive support and how it encourages voice behaviour in the tourism industry, and Lan et al.’s (contribution 3) study of taking-charge behaviours, which demonstrates that frontline hospitality employees are likely to take charge when they experience role clarity, job competence and supervisor developmental feedback, each point, in different ways, to the same broader conclusion: positive behaviour at work is cultivated through contexts that confer legitimacy, confidence, and support for initiative. These studies therefore shift attention away from the idea that positive organisational behaviour is merely a matter of individual willingness and address the conditions that make such behaviour possible and sustainable.

A related contribution of the Special Issue lies in its emphasis on work design and organisational systems as foundations of positive organisational functioning. Zang and Lyu’s (contribution 4) research on proactive customer service performance, showing that it is enhanced by high-commitment human resource systems and by strengthening employees’ sense of mission valence and work meaning, together with Yu et al.’s (contribution 5) findings on job crafting, indicating that a growth mindset predicts job crafting, particularly when employees experience job autonomy, show that employees are most likely to act proactively when organisational systems create the scope for agency, meaning, and self-directed action. These papers highlight that positive work behaviour is enabled through structures that provide autonomy, purpose, and alignment between organisational goals and employees’ sense of contribution.

Another strength of the collection is its treatment of psychological resources as central to both performance and wellbeing. Chompukum and Vanichbuncha’s (contribution 6) findings, which demonstrate that employee engagement and retention are promoted by psychological empowerment, alongside Cavallari et al.’s (contribution 7) analysis of emotional self-efficacy in healthcare settings, which indicates that it can buffer the impact of verbal aggression on psychological withdrawal and job satisfaction, underscore the importance of internal capacities that allow employees to remain engaged and effective even in demanding circumstances. These contributions suggest that positive organisational functioning often depends on resources that help employees navigate challenges, protect their wellbeing, and sustain constructive involvement in work.

The Issue also makes an important contribution by treating wellbeing as integral to positive organisational processes. Marzocchi et al.’s (contribution 8) paper positions flourishing both as an outcome of decent work and as a mechanism through which decent work supports performance over time. García-Selva et al. (contribution 9) similarly demonstrate that self-efficacy, perceived organisational support, and positive emotions are closely tied to work wellbeing, job satisfaction, and organisational commitment. Taken together, these studies strengthen the case for viewing wellbeing as embedded within organisational functioning rather than external to it.

Although fewer in number, the papers engaging with PWO 2.0 themes add a timely and necessary layer of complexity to the Special Issue. Chen and Zhang’s (contribution 10) analysis of after-hours work connectivity captures the ambivalence of digitally mediated work, showing that the same technological conditions can simultaneously expand control and performance while also generating anxiety. Barbu et al.’s (contribution 11) qualitative study complements this by bringing employees’ lived experiences of workplace technology into view, highlighting both its opportunities and tensions in relation to efficiency, collaboration, development, communication, and work–life balance. These papers point to the next frontier of the field: understanding how scholarship must adapt to a working world increasingly shaped by digital intensification, blurred boundaries, and shifting employee expectations.

Taken as a whole, the Special Issue suggests that the future of Positive Work and Organisations lies in integrative thinking. What emerges is a view of positive organisations as systems in which supportive conditions, personal capacities, and organisational practices combine to foster contribution, resilience, and flourishing. The broader message is therefore clear: organisations flourish when they attend not only to what employees do but also to the relational, structural, and psychological conditions that enable them to do it well and to thrive in the process.

## 7. Conclusions

Positive Work and Organisations is an intellectual reorientation and an applied agenda for bringing the realities of work into closer alignment with human flourishing and sustainable organisational functioning. This Special Issue has shown how PWO integrates insights from Positive Psychology and Management scholarship to illuminate the individual, relational, and systemic conditions under which people can thrive at work.

At the same time, the review of the field trajectory highlights a central tension: the field’s growth has been suppressed and accompanied by substantive critiques. These critiques matter not only academically but also strategically because they shape whether PWO is perceived as a rigorous, cumulative science capable of informing high-stakes organisational decisions or as a set of attractive ideas vulnerable to simplification and short-lived trend adoption. Addressing these challenges will require stronger theory, more rigorous and pluralistic methods, better replication and evaluation, and more credible science–practice partnerships.

Looking ahead, the future relevance of PWO will depend on its capacity to evolve alongside the changing nature of work. The PWO 2.0 agenda signals a shift towards greater engagement with technology, datafication, and AI-mediated working life, while retaining a commitment to human-centred design and ethical governance. If pursued effectively, this direction offers a route to renewed theoretical traction and practical impact. The next phase of the field is therefore likely to depend less on the introduction of new constructs and more on its ability to shape work as a developmental, relational, and ethical environment suited to the complexities of the 21st century.
